# Vitamin D Intake among Premenopausal Women Living in Jeddah: Food Sources and Relationship to Demographic Factors and Bone Health

**DOI:** 10.1155/2018/8570986

**Published:** 2018-03-19

**Authors:** Tahani A. Zareef, Robert T. Jackson, Abdulkareem A. Alkahtani

**Affiliations:** ^1^Department of Nutrition and Food Science, University of Maryland, College Park, MD 20742, USA; ^2^Department of Nuclear Medicine, King Abdulaziz Medical City, Jeddah, Saudi Arabia; ^3^College of Medicine, King Saud bin Abdulaziz University for Health Sciences, Jeddah, Saudi Arabia

## Abstract

**Background:**

Saudi women depend on food sources to maintain their serum 25(OH) D concentrations because covering by traditional clothing and time spent indoors limit their sun exposure. Little is known about vitamin D intake and its main food sources in Saudi Arabia. In addition, the association between vitamin D and calcium intake and bone mineral density (BMD) in young women is not well researched.

**Objectives:**

To assess the adequacy of vitamin D intake among Saudi women as compared to the estimated average requirements (EARs), to identify dietary vitamin D sources, to examine potential determinants of vitamin D intake, and to assess bone health and the association of calcium and vitamin D intake with BMD.

**Methods:**

This cross-sectional study was conducted in 257 premenopausal women aged 20–50 years in Jeddah, Saudi Arabia. Dietary vitamin D and calcium were assessed by the Semiquantitative Food Frequency Questionnaire. BMD was measured using dual-energy X-ray absorptiometry (DXA) in a subset of women (*n*=102) at the lumbar spine and femur neck.

**Results:**

Sixty-five percent of women were below the EAR for vitamin D, and 61% fell below the EAR for calcium. Dairy products, supplements, and fish contributed most to vitamin D intake. Increased age was an independent determinant of sufficient vitamin D intake (*p* < 0.001). The prevalence of osteopenia was 33% in the lumbar spine and 30% in the femur neck. There was a significant positive association between calcium intake and BMD at the lumbar spine (*p*=0.043) after controlling for body mass index and energy intake. Vitamin D intake was not significantly different between women with low and normal bone mass.

**Conclusion:**

Premenopausal women in Jeddah have insufficient vitamin D and calcium intakes. Public health strategies to improve nutrition in young women are needed, and expanding fortification programs to include all dairy products would be useful.

## 1. Introduction

Vitamin D is obtained through synthesis in the epidermis with exposure to ultraviolet (UVB) sunlight which converts 7-dehydrocholesterol to previtamin D3. It is naturally present in very few foods, mainly vitamin D3 (cholecalciferol) from animal sources including oily fish, egg yolks, veal, beef, and liver [1–3]. Vitamin D2 (ergocalciferol) comes from plant sources including sun-dried mushrooms [3].

Sunlight exposure, rather than diet, has been reported as the main source of vitamin D for the majority of the population [1, 2]. However, sun exposure has become a less viable source of vitamin D due to widespread use of sunscreen and a more sedentary indoor lifestyle [4] and for certain populations with limited sunlight exposure, such as veiled women [5]. Limited exposure to sunlight has consistently been identified as a main contributing factor for vitamin D deficiency (serum hydroxyvitamin D 25(OH) D < 50 nmol/L) in Saudi Arabia, particularly in women because the traditional clothing covers most of the body. Therefore, dietary intake represents the primary means of attaining vitamin D sufficiency when environmental, cultural, or physiological factors prevent adequate skin exposure to sunlight [1]. However, because there are few vitamin D sources in the diet, fortification of staple foods with vitamin D such as milk and margarine has become standard practice worldwide to help the population meet its need.

Fortification measures in Saudi Arabia are limited. Many Saudi dairy companies fortify milk and Laban (buttermilk) with vitamin D (400 IU/L). Fortification of other foods, such as cheese, cereal, and vegetable oils, is fairly low and variable in Saudi Arabia. Yet, even a national fortification program may not guarantee adequate vitamin D intake, particularly for at-risk populations with certain dietary patterns, such as low milk consumption [1]. In addition, it is not usually possible to achieve adequate vitamin D intake and maintain vitamin D status in at-risk groups without vitamin D supplements; therefore, a dietary supplement of vitamin D should also be considered when assessing sufficient vitamin D intake [5]. A limited number of studies in Saudi Arabia have documented a lack of consumption of dairy products and products supplemented with vitamin D [6]. No data are available, however, to examine dietary adequacy of vitamin D intake among premenopausal Saudi women. Furthermore, little is known about actual vitamin D intake in Saudi Arabia or the main food sources. No work has been done relating these intake levels to sociodemographic factors, which may help identify subpopulations at risk of inadequate vitamin D intake and potential vitamin D deficiency.

On the other hand, vitamin D and calcium are essential nutrients for maximizing peak bone and preventing bone loss, thus decreasing osteoporosis and the risk of fractures over the long term [7], both of which are growing public health and economic issues in Saudi Arabia. The prevalence of lumbar and femur osteopenia ranges from 7 to 43.4% and osteoporosis from 2.5 to 46.7% [8, 9]. Vitamin D deficiency due to limited sun exposure or inadequate amounts of dietary vitamin D decreases the efficiency of intestinal calcium absorption, resulting in increased parathyroid hormone (PTH) concentrations. Secondary hyperparathyroidism due to low levels of 25(OH) D may lead to increased bone loss, resulting in osteopenia, osteoporosis, and an increase in the risk of fractures in adults [10, 11]. Most Saudi studies investigating the role of nutrition in bone health have utilized only postmenopausal women [8, 12]. Bone mineral density (BMD) in premenopausal Saudi women has not been studied to a great extent. Young women are ideal candidates for investigating BMD research, as they can optimize their BMD and decrease their risk of fractures through dietary and lifestyle interventions while still being young [13].

Thus, the aim of this study was to assess the adequacy of vitamin D intake from food and supplements among premenopausal women in Jeddah as compared to the estimated average requirement (EAR) of 400 IU/day, to investigate the sources of vitamin D in their diet, and to examine potential determinants of vitamin D intake, including demographic factors. In addition, we evaluated the association between calcium intake from food and supplements, vitamin D intake from food and supplements, serum vitamin D levels, and serum parathyroid hormone levels with BMD in a subgroup of premenopausal women in Jeddah.

## 2. Materials and Methods

### 2.1. Study Design and Participants

A cross-sectional study of premenopausal Saudi women aged 20 to 50 years was conducted at King Abdulaziz Medical City (KAMC) in Jeddah, Saudi Arabia. Two hundred fifty-seven participants were selected from attendees of the primary care clinic at KAMC using systematic sampling. The sampling frame was derived from daily listing sheets, which include walk-in females who were seeking general outpatient services. A systematic sampling method was applied to the list of eligible women who were seen in the primary care clinic during the study period between December 2014 and April 2015. To implement the systematic sampling, a random number between one and three was generated each day. The random number determined which woman was chosen first from the list. Starting with the first woman chosen, then every third woman on the list was selected for potential inclusion in the sample. The exact process was repeated daily until the targeted sample size (*n*=257) was reached. Women were eligible for the study if they were citizens of Saudi aged 20 to 50 years. Women were excluded from the study if they were pregnant or lactating or postmenopausal (defined as amenorrhea for at least six months). Women with cancer, diabetes, hyperthyroidism or hypothyroidism, metabolic bone disease, malabsorption, and renal diseases, or those taking medications known to effect bone metabolism were also excluded from the study. To select participants for the DXA scan, each woman, chosen through the systematic sampling method described above, was asked to participate in this portion of the study until completion of the required sample for this subgroup (*n*=102) was achieved. Upon completion of the first visit, each woman was asked to return for a second visit, during which bone mineral density (BMD) measurements were taken via DXA scan.

### 2.2. Data Collection

#### 2.2.1. Anthropometric Measurements

Weight and height were measured. Body Mass Index (BMI) was calculated by dividing the weight (kilograms) by the square of the height (meters). Standard BMI cut points according to the World Health Organization (WHO) criteria [14] were used to categorize the weight status: underweight (>18.5 kg/m^2^), normal (18.5–24.9 kg/m^2^), overweight (25–29.9 kg/m^2^), and obese (≥30 kg/m^2^). Waist circumference (centimeters) was measured. According to the International Diabetes Federation abdominal obesity cut points for people in the Middle East (≥80 cm in women), waist circumference was dichotomized into normal (<80 cm) and abdominal obesity (≥80 cm) [15].

#### 2.2.2. Demographic Variables

Demographic variables included age, marital status, occupation status, monthly household income divided into categories (depending on the amount of Saudi Arabian riyal (1 SAR  =  0.266 USD)): (<4000, 4000–<8000, 8000–<15000, 15000–<25000, and ≥25000) [16], and residence (rented apartment, owned apartment, rented house, owned house, and others). Participants were asked about the number of years of formal education, and then, the level of education was categorized as follows: less than college, college graduate, and postgraduate. Participants were asked about the number of pregnancies and number of living children. Breastfeeding variables included the number of breastfed children and use of vitamin D supplementation during breastfeeding (yes/no).

#### 2.2.3. Dietary Calcium and Vitamin D Intake

Dietary intake of vitamin D per day was estimated using the Semiquantitative Food Frequency Questionnaire (SFFQ). The SFFQ used was a modified version of a previously validated questionnaire [17], which was also validated in United Arab Emirates and showed positive correlation between the SFFQ and 3-day diet food records (*r*=0.82, ⁡*p* < 0.001 and *r*=0.74, ⁡*p* < 0.001 for calcium and vitamin D, respectively) [18]. The modified version was designed to take into account the food composition for the Saudi population [19] and Arabian Gulf diet [20] that are considered to be excellent sources of calcium. Vitamin D values of some food items were obtained from the Arabian Gulf Food Composition Table such as meat and local fish consumed in this area. However, because vitamin D values for nonlocal foods such as tuna and salmon are not available in the Arabian Gulf Food Composition Table, the U.S. Department of Agriculture reference data [21] were used. Vitamin D values of supplements and local food items such as vitamin D-fortified milk and Laban drink (buttermilk) were obtained from food labels. The items in the SFFQ include natural food sources of vitamin D (e.g., tuna, salmon, meat, poultry, liver, and eggs), local, and imported vitamin D-fortified food and beverages available in the Saudi marketplace. Supplemental vitamin D and/or calcium and multivitamins were assessed by the SFFQ.

The selection of food items in the SFFQ was obtained and reviewed with the investigator using visual estimates (photographs) to help subjects estimate the average portion size. On the SFFQ, respondents were asked to record how often they consumed a single serving of each food listed during the previous month, with possible responses ranging from less than once per month to 2 or more per day. Vitamin D and calcium intakes were computed by multiplying the intake frequency by the nutrient content of the portion size of specific items. Intake of vitamin D and calcium was calculated by summing nutrient intake from diet and supplemental sources. Vitamin D and calcium intakes were examined in terms of absolute and energy-adjusted nutrients (intake per 1,000 kcal). Energy-adjusted values, termed “nutrient densities,” were computed as daily intake from diet and supplements divided by calories per day and multiplied by 1,000 [22, 23].

#### 2.2.4. Sun Exposure and Lifestyle Variables

The sun exposure recall questionnaire included details of the time of the day of sunlight exposure and length of exposure to sunlight during the previous month on weekdays and weekends. Cover status when they were outside in the public area was used to determine the area exposed to direct sunlight. Women were classified as either (1) covering the whole body including the face, (2) face covered but hands exposed, or (3) face and hands exposed. From the amount of clothing worn outside, the percentage of body surface area (BSA) exposed to sunlight was determined using an adjusted “rule of nines” (9% for the face, 1% for each hand, 9% for each arm, and 18% for each leg) used in clinical practice to estimate the burnt area of the skin [24]. A Sun Exposure Index (SEI) was calculated as the number of hours per week spent in direct sun without sunscreen protection multiplied by the fraction of BSA-exposed sunlight to determine the amount of sun exposure for each participant [24]. Life style factors included physical activity (PA) and smoking status (yes/no). PA was assessed using the Short Version of the International Physical Activity Questionnaire [25].

#### 2.2.5. Biochemical Analysis

Nonfasting venous blood samples were drawn for serum 25-hydroxyvitamin D 25(OH) D, parathyroid hormone (PTH), adjusted serum calcium (adCa), phosphate (P04), and alkaline phosphates (ALPs). Serum calcium was measured by Arsenazo III dye (Architect 16000, Abbott, USA). PTH was measured by a two-step sandwich immunoassay for the quantitative determination of intact PTH in human serum using CMIA technology (Architect 2000, Abbott, Germany). The PTH assay showed a detection sensitivity of ≤3.0 pg/mL, intra-assay CV was 12.9–6.1%, and interassay CV was 3.0–6.4%. The reference range for PTH in the study laboratory is 24–114 pg/mL. 25(OH) D in serum was determined by using a delayed one-step chemiluminescent microparticle immunoassay (CMIA) (Architect, Abbott, Germany). The laboratory undertaking the testing is a participant in the proficiency accuracy-based vitamin D since 2012. Intra-assay 25(OH) D coefficient of variation (CV) from daily quality control was 1.4–3.7%, and interassay CV was 2.6–4.6%. The fully automated architect assay is designed to have an imprecision of ≤10%.

The serum samples were taken in the laboratory in the primary care clinic; after blood collections, the samples were left for 10 min for clotting and then immediately centrifuged at 3000 rpm for 10 min. Then, the samples were aliquoted and frozen at −20°C until further analysis. All analyses were performed in the Pathology Laboratory at KAMC. Baseline serum 25(OH) D levels were classified into two categories according to the Institute of Medicine and WHO [11, 26]: vitamin D deficiency serum 25(OH) D < 50.0 nmol/L and sufficiency serum 25(OH) D ≥ 50 nmol/L.

#### 2.2.6. Measurement of Bone Mineral Density (BMD)

Bone mineral density (BMD) was measured using dual-energy X-ray absorptiometry (DXA) (Lunar Prodigy Advance, GE Medical Systems, Madison, WI 53718, USA) in a subgroup of 102 women at the lumbar spine (L1 to L4) and the neck of the femur. The precision of the measurements was 1.0–1.2% and 1.1–2.2% in the lumbar spine and the neck of the femur, respectively. DXA scans were performed in the Department of Radiology at KAMC. The BMD values at the hip were compared with peak bone mass for Caucasian female normative database from the National Health and Nutrition Examination Survey NHANES III data, as recommended by the International Society for Clinical Densitometry [27]. In addition, the Saudi Osteoporosis Society Committee released guidelines in 2015 stating that, in the absence of a local normative reference, the committee suggests to continue using the data from the United States because the NHANES III data are accurate and reliable and they seem to be best option until the local data become available [28]. BMD values were classified according to the WHO criteria: a low bone mass (osteopenia) if BMD T-score is <−1.0 or normal bone mass if BMD T-score is ≥−1.0 [29].

### 2.3. Ethics Statement

The research protocol and the consent documents were approved by the Ethics Committee of the Institutional Review Board (IRB) at the University of Maryland, College Park (no. 539327-3), and IRB at King Abdullah International Medical Research Center (KAIMRC), Jeddah, Saudi Arabia (no. RJ14/015/J). Participants were informed about the aim of the study, the clinical tests, and the type of interview.

### 2.4. Sample Size and Power Analyses

An a priori power analysis was conducted to determine the number of participants required to detect a small effect (*f*2 = 0.10) with power = 0.80, given the following testing parameters: a multiple forward stepwise regression with 16 predictors and conducted at *α* = 0.05. The analysis indicated that a sample size of 207 would detect a small effect given these parameters. The power analysis was conducted with G∗Power 3.1 software package [30]. To account for any potential loss of participants, an additional recruit of 20% was considered, and a total sample size was 250 women. To determine the required number of participants in the DXA subsample, the power analysis was also performed. Specifically, the sample size needed to detect the effect of a specific predictor (“serum vitamin D level”) with a power level of 0.80 and an alpha level of 0.05, using a multiple logistic regression with a binary response variable, assuming a moderate effect size (odds ratio = 2.50) and the squared multiple correlation coefficient between “serum vitamin D level” and other independent variables in the model = 0.3, was computed. The results indicate that assuming a medium effect size, a minimum of 76 subjects are required in order to achieve a power of 80% at the 0.05 level of significance.

### 2.5. Statistical Analysis

Data were analyzed using the Statistical Package for Social Science (SPSS) software version 22.0 (SPSS, Inc., Chicago, IL, USA). The characteristics of the study population were described through frequencies and percentages for categorical variables. For continuous variables, medians and interquartile ranges (IQRs) were used to summarize nonnormal data, and means ± standard deviations (SDs) were used to summarize data with normal distribution. The 257 women were divided into two groups: 167 women with insufficient vitamin D intake from food and supplement (ISVDI) (<400 IU/day) and 90 women with sufficient vitamin D intake from food and supplement (SVDI) (≥400 IU/day). Median anthropometric, dietary, and biochemical values between groups were compared using nonparametric Mann–Whitney *U* tests. A nonparametric Wilcoxon signed-rank test was used to determine if vitamin D and calcium intakes were statistically significantly different from the U.S. Estimated Average Requirement (EAR) of 400 IU/day and 800 mg/day, respectively. Percentages values between groups were compared using the chi-square tests. Kruskal–Wallis tests were performed to test for differences in intakes of vitamin D and calcium among age groups. Spearman rank correlations were used to assess the strength of the relationship of BMD at the lumbar spine and the femur neck with variables such as BMI, parity, and smoking, variables that were thought to influence BMD. Statistical significance was considered at *p* value < 0.05.

The forward stepwise multiple linear regression was conducted in order to investigate the relationship between the dependent variable, vitamin D intake (from food and supplement), and the independent variables, the sociodemographic and parity variables. The condition for entry at each iteration was *α* = 0.05, and the condition for predictor removal at each iteration was *α* = 0.10. In these analyses, the dependent variable vitamin D intake was log transformed since it was not normally distributed. The following independent variables were considered for entry in the stepwise regression: age, years of formal education, the number of children, income level, occupation, type of residence, marital status, and taken vitamin D supplement during the breastfeeding period. Note that age, years of formal education, and the number of children were continuous variables, and the remaining variables were categorical variables. Additionally, in order to keep the sample size as balanced as possible for each level of the categorical variables, some regrouping was implemented. The new categories for each categorical variable are as follows: household income level (less than 15000 versus 15000 and above), occupation (employed versus not employed), type of residence (owned versus not owned), marital status (married versus unmarried (single/separated/divorced)), and taken vitamin D supplement during the breastfeeding period (yes versus no). The assumptions for the regression model were evaluated using normal quantile-quantile (QQ) plots of the standardized residuals (the assumption of normality) and scatter plots with standardized residuals versus standardized predicted values (the assumption of homogeneous error variance). The assumptions of the regression model were satisfied.

Multiple logistic regressions were implemented to identify and analyze factors that influence BMD markers. Hence, the two dependent variables were BMD at the lumbar spine and BMD at the femur neck, both being binary response variables (low and normal). The independent variables being considered included vitamin D intake, calcium intake, serum 25(OH) D levels, and serum PTH level. The following variables served as possible confounding variables: BMI and energy intake. The Wald test was used to determine if an effect was statistically significant. Statistical significance was considered at *p* value < 0.05. The Hosmer–Lemeshow goodness-of-fit test was used to determine the model adequacy, and *p* value > 0.05 indicates good model fit. In this analysis, 88 subjects were used for logistic regression, after excluding subjects with missing values in the study variables.

## 3. Results

### 3.1. Characteristics of the Study Population

Demographic, anthropometric, dietary intake, and biochemical characteristics of the 257 premenopausal Saudi women aged 20–50 years, who were divided into two groups—insufficient vitamin D intake (ISVDI) (<400 IU/day) and sufficient vitamin D intake (SVDI) (≥400 IU/day)—are presented in Table 1. Participants with ISVDI were younger, less likely to consume vitamin D from dietary sources, and less likely to consume calcium from diet and supplements than participants with SVDI. A significantly higher percentage (95%) of vitamin D deficiency (serum 25(OH) D < 50 nmol/L) was observed in premenopausal women in the ISVDI group compared with 47% in the SVDI group (*p* < 0.001).

### 3.2. Vitamin D and Calcium Intake

The median daily intakes of vitamin D and calcium from all sources are presented in Table 2, for the whole sample and according to age groups. Overall, median daily vitamin D intake from diet and supplement was 236.4 IU/day, which is lower than the EAR; however, this difference was not statically significant (*p*=0.52) (Table 2). Approximately 97% of women fell below the EAR of vitamin D 400 IU/day based on their vitamin D intake from food alone (data not shown). When including supplements, more than half of women (65%) were below the EAR for vitamin D (Table 2). Thirty-seven percent of women reported using vitamin D supplements or multivitamins containing vitamin D in the past 30 days. Median vitamin D intake from food and supplement was significantly lower in young women aged 20–30 years compared with women aged 31–40 (*p*=0.029) or 41–50 years (*p* < 0.001). Additionally, a higher proportion of young women aged 20–30 years ate less than the EAR for vitamin D (20–30 = 74%; 31–40 = 57.5%; 41–50 = 35.5; *p* < 0.001). For calcium intake, median daily intake from diet and supplement was significantly lower (*p*=0.005) than the EAR (Table 2). Approximately 61% of women did not meet their EAR for calcium. About 15% of women reported using calcium supplements in the past 30 days. The daily intake of calcium from food and supplement showed a significant difference among age groups (*p* < 0.015). The median intake of calcium was significantly higher in women aged 41–50 years when compared to the other two younger age groups. A higher proportion of young women aged 20–30 and 31–40 years ate less than the EAR for calcium (20–30 = 66.7%; 31–40 = 58.9%; 41–50 = 35.5%; *p*=0.005). The intakes of vitamin D and calcium were found to be strongly correlated (*r*_2_=0.73, ⁡*p* < 0.001) in this study population.

The sources of vitamin D (% of total vitamin D intake) in 20- to 50-year-old women are presented in Table 3. For women between 20 and 30 years, the top three vitamin D sources were dairy products (38.4%), supplement (25.2%), and fish (12.1%); for women between 31 and 40 years, the top three vitamin D sources were supplement (37.9%), dairy products (32.7%), and fish (12.5%); for women between 41 and 50 years, the top three vitamin D sources were supplement (43.8%), dairy products (35.9%), and fish (7.9%).

### 3.3. Association of Vitamin D Intake with Sociodemographic Factors

Evaluating the correlations between vitamin D intake and sociodemographic factors, we found that vitamin D intake was statistically significant and positively correlated with age (*r*_2_=0.27, ⁡*p* < 0.001), marital status (*r*_2_=0.14, ⁡*p*=0.03), gravidity (*r*_2_=0.18, *p*=0.004), parity (*r*_2_=0.14, *p*=0.02), and BMI (*r*_2_=0.13, *p*=0.041). The results of the forward stepwise regression suggest that there was a statistically significant relationship between log-transformed vitamin D intake (diet and supplement) and age (*B*=0.039, *p* < 0.001) (Table 4). The *R*^2^ = 0.10 indicates that 10% of the variation in the dependent variable can be accounted for by the model. There was no statistically significant relationship between log-transformed vitamin D intake and the other independent variables, including the income level, years of formal education, occupation, type of residence, marital status, the number of children, and taken vitamin D supplement during the breastfeeding period.

### 3.4. Bone Mineral Density (BMD) Measurements

Out of 257 premenopausal women, 102 women agreed to undergo DXA scan. Median serum 25(OH) D levels were low (median 40 nmol/L). Of the women in this analysis, there was a high proportion, 77.5% of the women, with vitamin D deficiency (25(OH) D < 50 nmol/L), and of these, approximately 14% had severe vitamin D deficiency (25(OH) D < 25 nmol/L). Elevated PTH was seen in 16% (14/88) of the women (PTH mean 160.7 ± 41 pg/mL, laboratory reference range 24–114 pg/mL); among those with elevated PTH, 78.6% (11/14) women had vitamin D deficiency (25(OH) D < 50 nmol/L). The prevalence of osteopenia was 33% and 30% in the lumbar spine and the neck of the femur, respectively. Of the 102 women, 55 (54%) women did not suffer from osteopenia in both sites, whereas 18 (17.6%) women had osteopenia in both sites. The remaining 29 women (28.4%) had osteopenia in one of the sites. Approximately 45.5% (5/11) of our participants who experienced vitamin D deficiency and elevated PTH had lower BMD at either site.

### 3.5. Association of Vitamin D Intake, Calcium Intake, Serum 25(OH) D, and Serum PTH with Bone Mineral Density (BMD)

In univariate analysis (Spearman correlation), the measures of BMD at the lumbar spine and the femoral neck showed significant positive relationship with anthropometric measurements. There was a significant positive correlation between height and BMD (*r*_2_=0.27, *p*=0.006 at the lumbar spine and *r*_2_=0.34, *p* < 0.001 at the femoral neck). Similarly, there was a significant positive correlation between BMD at the lumbar spine and at the femoral neck and weight, and BMI, (*r*_2_=0.44, *p* < 0.001 and *r*_2_=0.37, *p* ≤ 0.001, resp.). No significant association was found between BMD and age, sun exposure, physical activity, parity, and smoking.  Table 5 shows the results of the logistic regression with the dependent variable BMD at the lumbar spine. The analysis results suggested that there was a statistically significant relationship between BMD at the lumbar spine and calcium intake (*χ*^2^(1, *N*=88)=4.100, *p*=0.043). The odds ratio was 1.002 with 95% confidence = (1.000, 1.005). There was a statistically significant relationship between BMD at the lumbar spine and BMI (*χ*^2^(1, *N*=88)=6.06, *p*=0.014). The odds ratio was 1.117 with 95% confidence = (1.023, 1.219). There was a statistically significant relationship between BMD classification at the lumbar spine and energy intake (*χ*^2^(1, *N*=88)=7.362, *p*=0.007). The odds ratio was 0.997 with 95% confidence = (0.995, 0.999). There was no statistically significant relationship between BMD classification at the lumbar spine and serum 25(OH) D, serum PTH, and vitamin D intake. At the femur neck, there was no statistically significant relationship between BMD classification and calcium intake, serum 25(OH) D, serum PTH, and vitamin D intake.

## 4. Discussion

### 4.1. Dietary Adequacy of Vitamin D and Calcium

The results of the current study showed that this sample of premenopausal women (aged 20–50 years) in Jeddah did not consume sufficient amounts of dietary vitamin D and calcium to meet the EAR for this age group. The low vitamin D intake observed in the present study is consistent with that which has been previously reported in the Middle East. In a study of Bahraini women (*n*=325, mean age = 28 years), Alhaddad et al. reported a mean dietary intake of 187 IU/day of vitamin D, which was insufficient to meet bodily requirements [31]. Production companies in Bahrain voluntarily fortify dairy products, while milk and dairy products imported from Saudi Arabia are also fortified with 400 IU/L of vitamin D. It appears that the current fortification measures used in Saudi Arabia and Bahrain are not sufficient to meet the EAR for vitamin D intake in women in both countries. However, the overall estimates of this vitamin D intake still appear to be much higher than those previously reported in other Middle Eastern countries, in which there is an absence of vitamin D fortification in milk-derived food products. Gannagé-Yared et al. reported that the mean daily vitamin D intake of Lebanese women (*n*=214, aged 20–50 years) was 89.0 IU/day from food sources [32], whereas the vitamin D intake from food sources (excluding supplement use) by the women in the current study was 141 IU/day. The absence of vitamin D fortification in milk-derived products, such as Laban and yogurt, which are highly consumed in Lebanon and Saudi Arabia, may explain these differences.

### 4.2. Vitamin D Sources and Serum 25(OH) D Concentrations

In analyzing the various vitamin D sources, we found that women have a high consumption rate of fortified dairy products, which explains the importance of dairy products, particularly Laban and yogurt, as major sources of vitamin D. In addition, vitamin D supplements and canned fish, such as tuna, are considered to be good sources of this vitamin among women in Jeddah. An international comparison of vitamin D intake and its main sources of published information with regard to Australia, Japan, Norway, Lebanon, Taiwan, the US, and the UK, along with the results of the current study, are summarized in Table 6 [1, 3, 5, 32]. Differences in food-fortification policies or food composition between countries may have led to differences in vitamin D intake and in the main contributory food sources [33]. Saudi Arabian dietary patterns are comparable to those of the Lebanese population (Table 6); Saudi and Lebanese individuals have a high consumption rate of dairy products (36.5% and 30.2%, resp.). However, the consumption of canned fish, such as tuna, meat, and organ meat, such as liver, is higher in Lebanese individuals. The observed percentage differences between the studies may be explained by the proportion (37%) of women who took vitamin D supplements in the present study, which contributed 31% of their vitamin D intake; these supplements were an exclusion criterion in the Lebanese study. In the current study, almost 37% (*n*=96) of the women took vitamin D supplements during the last 30 days; of these, 52% (*n*=50) took single or multiple high doses, ranging from 15,000 to 50,000 IU. Approximately 30% (*n*=29) of the women took high-dose vitamin D2 and 22% (*n*=21) took high-dose vitamin D3, while the remaining 48% (*n*=46) of the women took vitamin D3 alone or in combination with calcium or other multivitamins that contained 400–4000 IU vitamin D3 per daily dose.

Vitamin D intake from food and supplements was associated with improved vitamin D status among women in the present study. Women whose vitamin D intake was below the EAR had a significantly higher percentage (95%) of vitamin D deficiency than those whose intake was above the EAR (47%; *p* < 0.001). Some studies have reported that participants' vitamin D intake is highly correlated with their serum 25(OH) D concentrations, and that this relationship may become stronger as the influence of solar ultraviolet B radiation weakens, particularly among people with limited sun exposure [34]. The median duration of sunlight exposure for women in the current study was short (9.4 min/day), which reflects the absence of an important source of vitamin D. Our data support the fact that sufficient vitamin D intake from food and supplements is important, and we found a statistically significant and positive association between such intake and serum 25(OH) D concentrations (*r*_2_=0.60, *p* < 0.001). Furthermore, Saudi women are dependent on food sources, rather than sun exposure, to maintain their serum 25(OH) D concentrations. This is partly because their fully covering traditional clothing limits exposure to the sun and partly due to the fact that they tend to spend more time indoors than outdoors because of the hot climatic conditions that prevail for most of the year.

Sufficient vitamin D intake among premenopausal women in Jeddah is unlikely to be achieved through dietary sources alone. Although vitamin D-fortified food may help to maintain vitamin D status in the general population, it may not be effective in correcting vitamin D deficiency in at-risk groups [5]. In 2014, the Saudi Food and Drug Authority (SFDA) issued a mandatory circular regarding vitamin D fortification that manufacturers were to adopt within a six-month period and continue to adhere to. The standards for this vitamin D fortification policy were developed by the Gulf Standard Organization and mandated that vitamin D should be added to whole milk, skimmed milk, and Laban at a level of 400 IU/L and to yogurt at a level of 40 IU/100 g [35]. However, our product label analysis indicated that there are still several companies that have not fortified their products with vitamin D despite the SFDA policy. In light of this, we recommend that the SFDA implements an appropriate monitoring program to ensure that the fortification policy is followed. Vitamin D supplements significantly improved dietary intake in the current study; 86.5% of the women who took a vitamin D supplement met the EAR of vitamin D, compared to 4.3% of the women who did not take a supplement of this kind (*p* < 0.001). In addition, 51% of the women who took a vitamin D supplement achieved a sufficient serum vitamin D level (25(OH) D ≥ 50 nmol/L), compared to 4.5% of the women who did not take a supplement (*p* < 0.001). Therefore, it is important to consider supplementation as an effective approach in helping to ensure sufficient vitamin D intake and to address vitamin D deficiency among women in Jeddah.

### 4.3. Vitamin D Intake and Sociodemographic Factors

Multiple linear regression analysis showed that only age was an independent predictor of vitamin D intake in premenopausal women in Jeddah. Previous studies on this topic are limited; none have investigated the demographic determinants of vitamin D intake, particularly among nonpregnant women. A recent study conducted in 68,447 pregnant Danish women found a lower level of sufficient vitamin D intake from diet and supplements, suggesting that young women may be less conscious of health matters compared to older women [36]. In the present study, we observed a lower level of sufficient vitamin D intake (≥400 IU/day) among young women (20–30 years) compared to middle-aged women (31–50 years). These differences may be explained by the higher consumption of vitamin D-rich food in middle-aged women, compared to young women (median 185.2 IU/1000 kcal/day versus 157.4 IU/1000 kcal/day, *p*=0.04), and a higher consumption of vitamin D-containing supplements (47.1% versus 30.7%, *p*=0.01). A high prevalence of vitamin D deficiency was also observed in young women, compared to middle-aged women (83% versus 70%, *p*=0.02). The results of the present study suggest that young women at greatest risk of vitamin D deficiency were consuming the lowest amount of vitamin D from dietary and supplementary sources, which confirms the results of previous reports. In a study conducted in healthy young Canadian women (*n*=107, aged 18–30 years), Gozdzik et al. underlined the fact that vitamin D intake was low among those who were at greatest risk of vitamin D insufficiency (25(OH) D < 75 nmol/L) [37]. In the present study, the importance of dietary supplements as a source of vitamin D increased from 25% at the age of 20–30 years to 44% at the age of 41–50 years.

In the bivariate analyses, being married and multiparty was positively associated with vitamin D intake. Our finding is inconsistent with the results of a previous study conducted in a Danish birth cohort [36], which showed that the use of vitamin D supplementation was inversely associated with parity and that multiparous women tended to have a lower overall vitamin D intake. An increased adherence to vitamin D and calcium supplements in the present study was more likely in women who more often attended antenatal care, where they received supplements. Some previous studies found that high household income and education were strongly associated with increased vitamin intake from food [4]. Although this difference was not observed in the present study, we found that women with a high household income (≥15000 SR) were more likely to consume vitamin D supplements than women with a low household income (<15000 SR) (47.2% versus 32.2%, *p*=0.02), which may explain the higher serum 25(OH) D concentrations that were found in women with high household income compared to those with low household income (median 25(OH) D: 38.1 nmol/L versus 33.2 nmol/L, *p*=0.035). This relationship has also been previously found. McCormack et al. investigated the prevalence of vitamin D supplement use in Canadians aged 45 years and over who participated in the Healthy Aging module of the Canadian Community Health Survey from 2008 to 2009 [38]. They found that vitamin D supplement use increased with household income and asserted that this may have been because individuals with low household income consider taking the supplements to be less important, particularly when faced with a shortage of food and money.

### 4.4. Dietary Vitamin D and Calcium Intake, Serum 25(OH) D, PTH, and Bone Health

The prevalence of osteopenia found in the present study is in accordance with the findings of other studies that were conducted in Saudi Arabia. For example, Youssef found that the prevalence of osteopenia was 39.7% in female students (aged 17–25 years, *n*=267) in Makkah [39]. The studies conducted in Saudi Arabia have shown that the prevalence of osteoporosis among postmenopausal women is higher than that observed in Western countries [28]; this prevalence ranged from 23% to 44.5% across different regions of the country [40, 41]. In the present study, we evaluated the relationship between dietary intake of calcium and vitamin D and serum 25(OH)D and serum parathyroid hormone PTH in women with low and normal BMD. The findings are in accordance with the generally accepted risk factors for low bone mass, namely, dietary calcium intake [7] and BMI [42, 43], whereas vitamin D intake, serum 25(OH) D, and PTH are not significant predictors of BMD in premenopausal women in this study. The observed shortfall in calcium intake below the EAR for more than half of the participants is in accordance with the results of other studies conducted in Saudi Arabia. Youssef found that the mean calcium intake of a group of female students (*n*=267, aged 17–25 years) in Makkah was 559.3 ± 32.2, with approximately 86.1% of the sample consuming less than 800 mg/day of calcium. Different types of dietary assessment methods may explain the observed differences between our study and the one conducted in Makkah [39]. The calcium intake from diet and supplements in our study was assessed using a Semiquantitative Food Frequency Questionnaire, while calcium intake was assessed by 3-day recall in the investigation conducted in Makkah. It has been found that when a calcium-rich food frequency questionnaire is compared with 3-day recall, participants may identify foods in the questionnaire but may not happen to have consumed those foods on the specific day of recall [44].

In the present study, calcium intake insufficiency was more pronounced in women with low lumbar spine bone mass than in women with normal bone mass; we observed a statistically significant but weak relationship between lumbar spine BMD and calcium intake. The correlation with dietary calcium has not been consistently reported. A number of epidemiological studies have failed to show positive associations between calcium intake and BMD [12, 42, 45], while others showed a positive one [7, 39]. Despite the well-known effects of calcium and vitamin D, which are essential nutrients for maximization of peak bone mass, this increase in bone density can prevent bone loss and thus reduce the risk of osteoporosis and fracture later in life [7]. Our study showed that current calcium intake had a very small effect on lumbar spine BMD but did not affect femoral neck BMD in the study population. Reviewing previous studies on this subject, it would appear that BMD is a complex parameter with wide variation among individuals' genetic factors, lifestyle and physical exercise, body weight and composition, and hormonal status [46], and each of these factors plays a significant role in adult bone health. Therefore, the influence of factors other than calcium on BMD could partially explain the weak or lack of correlation between current calcium intake and BMD in our study. However, this does not negate the potential beneficial effects of adequate calcium and vitamin D doses during childhood and adolescence, the period of peak acquisition, on bone mass [45–47]. It should also be noted that our study assessed current dietary calcium and vitamin D intake in premenopausal women without considering the women's dietary calcium and vitamin D intake earlier in their lives during childhood and adolescence, which positively influences BMD values in adults. This factor may contribute to the discrepancy between current calcium intake and BMD and thus possibly affect the outcome of our study [46, 47]. Low vitamin D intake was also observed in the present study; over half of the women had a lower intake than the EAR. Vitamin D insufficiency was similar in women with low and normal bone mass, and thus, this may have had no major effect on the outcome of this research. This finding is consistent with those of previous studies [12, 45], which reported no significant association between vitamin D intake and BMD measurements at the lumbar spine or hip in women. This confirms the need for more intense strategies to inform young women of the importance of sufficient dietary intake of vitamin D and calcium in optimizing bone health. Nevertheless, there is a need for a longitudinal study of the effects of prolonged dietary and supplementary vitamin D and calcium intake on BMD, targeting at-risk groups.

A low serum 25(OH) D concentration is considered to be an important risk factor for low BMD, and previous studies have shown positive correlations between serum 25(OH) D and BMD at the lumbar spine and at the neck of femur in premenopausal women [42]. However, other studies in premenopausal [48] and postmenopausal [8, 12] women have not supported this association. In the present study, we did not observe a significant association between serum 25(OH) D and BMD at the lumbar spine or at the neck of femur. Based on previous studies, both Saudi and international, it would appear that there are a number of variables which have an impact on the presence or absence of a correlation between vitamin D status and BMD. These include differences in population, age groups, geographical location, hormonal status, and the sites of the body studied. More important is the fact that, with a cross-sectional study like this one, serum 25(OH) D levels depict the current vitamin D status of an individual, while BMD indicates bone mineral accrual over a period of time depending upon a variety of other factors [49, 50]. In contrast, an inverse association between serum 25(OH) D and intact PTH was evident in women with serum vitamin D < 50 nmol/L (*r*=−0.27, *p*=0.03). The association between PTH and BMD has not been consistently observed. Some studies have shown that elevated serum PTH levels are significantly associated with lower BMD [51], while others have failed to demonstrate any significant relationship [12]. In the present study, the inverse association between PTH and BMD at the lumbar spine and at the neck of femur did not reach statistical significance. However, 7% of our participants who experienced vitamin D deficiency and elevated PTH had lower BMD at one of the two sites. These findings reveal the need for intervention and educational campaigns to improve low bone mass in young women, as, when left untreated, it may lead to osteoporosis and fractures in the long term. PTH and 25(OH) D are the major hormonal regulators of calcium homeostasis. It has been shown that when serum 25(OH) D is 50 nmol/L or lower, there is a significant decrease in intestinal calcium absorption, and that this decrease is associated with elevated PTH [52]. PTH maintains calcium homeostasis by enhancing tubular reabsorption of calcium and stimulates the kidney to activate 25(OH) D to 1,25(OH) D. Elevated PTH also stimulates the process of dissolving the bone matrix to liberate minerals, leading to osteopenia and osteoporosis and increasing the risk of fracture [52]. Body weight is an important risk factor for osteoporosis. In the present study, we found a strong positive relationship between BMI and BMD at both the lumbar spine and the neck of femur. Women with higher BMI tended to have higher BMD, and this positive association has previously been well documented [42, 43, 48].

The present study provides new insights regarding daily vitamin D intake and examined the food sources that make the biggest contribution among premenopausal women in Jeddah and the subgroup most vulnerable to insufficient vitamin D intake. In addition, this study is the first assessment of dietary intake of vitamin D and calcium among premenopausal women in Jeddah and its association with the bone health parameter, BMD. However, we must consider its limitations. First, SFFQ may not be the most accurate tool for use in the assessment of dietary sufficiency; thus, dietary vitamin D and calcium intake may be subject to measurement error. However, we attempted to minimize this error by using an interviewer to administer the questionnaire to help participants to accurately estimate portion sizes, rather than using a self-reported questionnaire. Second, the use of Caucasian normative data for peak adult bone mass may have led to overdiagnosis of the prevalence of osteopenia. Standardization of BMD by DXA to a database that is culturally specific for Saudi Arabia is necessary and recommended to ensure reliable interpretation of the individual DXA data and to capture those women at risk. Third, although the participants included in the present study were young and healthy, our results are limited to premenopausal women attending the primary health center at King Abdulaziz Medical City in Jeddah and not to all women residing in Jeddah or in other regions of Saudi Arabia.

## 5. Conclusion

Insufficient intake of vitamin D and calcium is a growing concern for premenopausal women in Jeddah. Our data indicated that vitamin D intake was particularly low among young women who were at greatest risk of vitamin D deficiency. This study supports the call for intense public health strategies to improve nutrition among young women. Such strategies should start in adolescence, before peak bone mass is reached, to help provide diets rich in calcium and vitamin D and promote physical activity. Dairy products provide the most calcium and vitamin D in the average diet; it is important for girls to meet the recommended intake of dairy products during peak height velocity [53]. We suggest that young women should be encouraged to select products fortified with vitamin D. Furthermore, lactose-intolerant women should be educated to make appropriate dietary substitutions, such as well-tolerated dairy products like Laban and yogurt. In addition, it would be beneficial to introduce new vitamin D-fortified products to the Saudi market, especially flavored yogurt, which is regularly consumed by young women like those in our study population. Toward this goal, we recommend that the SFDA encourages manufacturers to fortify such new products, which may help young women achieve adequate levels of vitamin D. In addition, we recommend that the SFDA website provides education on products fortified with vitamin D and the benefits of maintaining adequate nutrition. However, in spite of the known benefits of vitamin D fortification to the health of the general population, consumers of fortified products should be studied to identify any negative health effects that excessive vitamin D consumption might cause. Therefore, we strongly recommend future studies on the effects of this vitamin D fortification scheme on health outcomes in the general population [54, 55].

## Figures and Tables

**Table 1:** Demographic, anthropometric, and clinical characteristics of Jeddah premenopausal women (20–50 years) with sufficient vitamin D intake versus insufficient vitamin D intake^a^(*n*=257).

Characteristics	Insufficient vitamin D intake (*n*=167), median (P25–P75)	Sufficient vitamin D intake (*n*=90), median (P25–P75)	*p* value
Age (years)	27.0 (23.0–32.0)	31.0 (26.5–39.0)	<0.001^∗^^b^
BMI (kg/m^2^)	25.3 (22.1–29.3)	27.2 (22.6–32.1)	0.09^b^
Vitamin D intake (IU/day) (diet and supplement)	159.1 (90.3–228.7)	1630.2 (649.5–1842.4)	<0.001^∗^^b^
Dietary vitamin D intake (IU/day)	132.1 (76.4–210.2)	157.6 (91.5–255.6)	0.05^∗^^b^
Vitamin D intake from fish (IU/day)	15.6 (4.9–31.5)	21.8 (9.4–43.8)	0.04^∗^^b^
Calcium intake (mg/day) (diet and supplement)	665.2 (413.7–912.5)	811.3 (572.8–1089.0)	0.02^∗^^b^
Serum 25(OH) D (nmol/L)	29.5 (23.2–35.2)	51.4 (41.1–68.9)	<0.001^∗^^b^
BMI (kg/m^2^)	*n* (%)	*n* (%)	
Nonobese (<25 kg/m^2^)	80 (69)	36 (31.0)	0.22^c^
Obese (≥25 kg/m^2^)	87 (61.7)	54 (38.3)	
Serum 25(OH) D (nmol/L)			
Deficiency (<50 nmol/L)	152 (78.4)	42 (21.6)	<0.001^∗^^c^
Sufficiency (≥50 nmol/L)	8 (14.3)	48 (85.7)	
Education status			
Less than college	50 (67.6)	24 (32.4)	0.72^c^
College graduate	103 (63.2)	60 (36.8)	
More than college	14 (70.0)	6 (30.0)	
Income level (SR per month)			
<15000	97 (67.8)	46 (32.2)	0.15^c^
≥15000	52 (58.4)	37 (41.6)	

BMI, body mass index; WC, waist circumference; P25–P75, 25th percentile and 75th percentile. ^a^Sufficient vitamin D intake consumed ≥400 IU/day and insufficient vitamin D intake consumed <400 IU/day. ^b^The nonparametric Mann–Whitney *U* test was performed. ^c^The chi-square test was performed. ^∗^Significance at the <0.05 level.

**Table 2:** Vitamin D and calcium intake and percent below the EAR among Jeddah premenopausal women residents (*n*=257) stratified by age.

	Total (*n*=257)	*p* value^a^	20–30 years (*n*=153)	31–40 years (*n*=73)	41–50 years (*n*=31)	*p* value^b^
Vitamin D intake from diet and supplement (IU/day)						
Mean + SD	643.4 + 873.1		488.2 ± 718.7	854.5 + 1060.3	912.3 + 946.8	
Median (IQR: P25–P75)	236.4 (114.4–748.4)	0.52	206.8 (99.5–403.9)	276.9 (124.6–1632.1)	556.1 (228.9–1693.8)	<0.001^∗^
*n* (%) below EAR (400 IU/day)^c^	167 (65)		114 (74)	42 (57.5)	11 (35.5)	<0.001^∗^
Calcium intake from diet and supplement (mg/day)						
Mean + SD	770.9 + 421.9		725.2 ± 378.1	788.1 + 475.9	955.8 + 448.2	
Median (IQR: P25–P75)	702.7 (469.6–981.2)	0.005^∗^	681.3 (440.1–925.6)	713.7 (416.4–989.1)	962.8 (626.3–1100.0)	0.015^∗^
*n* (%) below EAR (800 mg/day)^c^	156 (61)		102 (66.7)	43 (58.9)	11 (35.5)	0.005^∗^

EAR, estimated average requirement; P25–P75, 25th percentile and 75th percentile; SD, standard deviation. ^a^The Wilcoxon signed-rank test was performed to compare differences between median nutrient intake and EAR. ^b^The nonparametric Kruskal–Wallis test was performed to compare differences in intake among age categories. ^c^Adequacy was determined using the estimated average requirement levels of 400 IU/day for vitamin D and 800 mg/day for calcium for women aged 19–50 years. ^∗^Significance at the <0.05 level.

**Table 3:** Sources of vitamin D intake in 20- to 50-year-old Jeddah premenopausal women and percentages of total vitamin D intake.

Vitamin D sources (IU/day)		Age category		
	Total (*n*=257)	20–30 years (*n*=153)	31–40 years (*n*=73)	41–50 years (*n*=31)
Supplement	31.0	25.2	37.9	43.8
Milk, Laban, yogurt	36.5	38.4	32.7	35.9
Cereal	1.1	1.6	0.5	0.3
Food made with milk	3.1	3.8	2.3	1.3
Fish	12.2	12.1	12.5	7.9
Meat	8.4	9.4	7.8	5.0
Egg	6.9	7.7	5.9	5.4

Foods that contributed 1% or more of total vitamin D intake are shown in the table.

**Table 4:** Sociodemographic factors associated with log-transformed vitamin D intake in forward stepwise regression for Jeddah premenopausal women (aged 20–50 years) residents.

	Unstandardized coefficients		Standardized coefficients		
	*B*	SE	Beta	*t*	*p* value
Intercept	4.551	0.323		14.074	<0.001^∗^
Age	0.039	0.010	0.240	3.754	<0.001^∗^

Dependent variable = log-transformed vitamin D intake from food and supplement; *n*=257; *B* = unstandardized regression coefficient; SE = standard error; beta = standardized regression coefficient; *t* = *t*-statistic. ^∗^Significance at the 0.05 level.

**Table 5:** Logistic regression analysis for association of vitamin D and calcium intake, serum 25(OH) D, and serum PTH with bone mineral density at the lumbar spine for Jeddah premenopausal women.

Variables	Wald	*p* value	OR	95% CI	
				Lower	Upper
Vitamin D intake	0.504	0.478	1.000	0.999	1.000
Calcium intake	4.100	0.043^∗^	1.002	1.009	1.005
Serum 25(OH) D	0.154	0.695	0.995	0.971	1.019
PTH	0.145	0.703	0.997	0.984	1.011
BMI	6.069	0.014^∗^	1.117	1.023	1.219
Energy intake	7.362	0.007^∗^	0.997	0.994	0.999
Constant	0.019	0.889	0.775		

Wald = Wald chi-square statistic; OR = odds ratio; CI = confidence interval. ^∗^Significance at the 0.05 level.

**Table 6:** The average dietary intake of vitamin D and main food sources of selected countries including the current study.

Country	Women age (years)	Vitamin D intake from food (IU/day)	Vitamin D intake from food and supplement (IU/day)	Main sources (top three)
Current study	20–50	141	236.4	Dairy products (36.5%), supplements (31%), fish (12.2%)
Australia [5]	Adult women	80–96		Margarine (48%), canned fish (16%), eggs (10%)
Japan [1]	≥40	284		Fish (91%), eggs (3%), meat (2%)
Lebanon [32]	30–50	89		Milk (30.2%), meat (28%), fish (25.5%)
Norway [1]	25–69		236	Supplements (49%), fish (26%), margarine (23%)
Taiwan [3]	19–44	154	176	Fish (39%), dairy products (14%), supplements (12%)
United Kingdom [1]	35–49		148	Supplements (34%), fish (19%), cereal (16%)
United States [1]	20–49		293	Supplements (40%), milk (39%), cereal (3%)

IU = international unit. The table format and information are adapted from *Vitamin D Intake and Its Food Sources in Taiwanese* by Lee et al. [3].
